# Phage φAB6-Borne Depolymerase Combats *Acinetobacter baumannii* Biofilm Formation and Infection

**DOI:** 10.3390/antibiotics10030279

**Published:** 2021-03-09

**Authors:** Md. Shahed-Al-Mahmud, Rakesh Roy, Febri Gunawan Sugiokto, Md. Nazmul Islam, Ming-Der Lin, Ling-Chun Lin, Nien-Tsung Lin

**Affiliations:** 1 Master Program in Microbiology and Immunology, School of Medicine, Tzu Chi University, No. 701, Sec. 3, Zhongyang Rd., Hualien 97004, Taiwan; shahed.shuvo16@gmail.com (M.S.-A.-M.); 106329105@gms.tcu.edu.tw (F.G.S.); mdinazmul@gmail.com (M.N.I.); 2 Institute of Medical Sciences, Tzu Chi University, No. 701, Sec. 3, Zhongyang Rd., Hualien 97004, Taiwan; rakeshroy1803@gmail.com; 3 Department of Molecular Biology and Human Genetics, Tzu Chi University, No. 701, Sec. 3, Zhongyang Rd., Hualien 97004, Taiwan; mingder@gms.tcu.edu.tw

**Keywords:** *Acinetobacter baumannii*, phage, tailspike protein, depolymerase, Foley catheter, capsular polysaccharide

## Abstract

Biofilm formation is one of the main causes of increased antibiotic resistance in *Acinetobacter baumannii* infections. Bacteriophages and their derivatives, such as tail proteins with depolymerase activity, have shown considerable potential as antibacterial or antivirulence agents against bacterial infections. Here, we gained insights into the activity of a capsular polysaccharide (CPS) depolymerase, derived from the tailspike protein (TSP) of φAB6 phage, to degrade *A. baumannii* biofilm in vitro. Recombinant TSP showed enzymatic activity and was able to significantly inhibit biofilm formation and degrade formed biofilms; as low as 0.78 ng, the inhibition zone can still be formed on the bacterial lawn. Additionally, TSP inhibited the colonization of *A. baumannii* on the surface of Foley catheter sections, indicating that it can be used to prevent the adhesion of *A. baumannii* to medical device surfaces. Transmission and scanning electron microscopy demonstrated membrane leakage of bacterial cells treated with TSP, resulting in cell death. The therapeutic effect of TSP in zebrafish was also evaluated and the results showed that the survival rate was significantly improved (80%) compared with that of the untreated control group (10%). Altogether, we show that TSP derived from φAB6 is expected to become a new antibiotic against multi-drug resistant *A. baumannii* and a biocontrol agent that prevents the formation of biofilms on medical devices.

## 1. Introduction

The Gram-negative bacterium, *Acinetobacter baumannii* is an opportunistic pathogen that frequently causes infections associated with medical devices, e.g., vascular catheters, cerebrospinal fluid shunts, or Foley catheters and is one of the most common bacteria linked to infections associated with intensive care units [1]. In clinical settings, *A. baumannii* has become a nosocomial pathogen with alarming increases in drug resistance rates. Undoubtedly, a capsular polysaccharide (CPS) covering *A. baumannii* is one of the key components for biofilm formation and crucial for its survival in adverse environments, pathogenicity and resistance to antimicrobial compounds [2,3]. It was reported in one study that more than 60% of *A. baumannii* isolates from clinical samples formed biofilms and these isolates were linked mainly with device-associated infections [4]. Biofilm can be usually defined as a surface-attached microbial community in which bacterial cells adhere to each other within a self-produced matrix of extracellular polymeric substances, including polysaccharides, proteins, nucleic acids, lipids, water and mineral ions, which protect them from the external environment. This complex structure makes the eradication of biofilm-associated bacteria from hospital environments almost impossible and confers high resistance to antibiotics and disinfectants. Several studies have demonstrated that biofilm formation facilitates the survival and persistence of *A. baumannii* on abiotic hospital surfaces, resulting in tenacious and recurring nosocomial infections and is also a major cause of antibiotic resistance and strong survival adaptability [4,5,6,7]. Therefore, novel methodologies are needed to control this evolving pathogen.

Bacteriophages (phages) are natural predators of bacteria that can kill their hosts after infection. The idea of using lytic phages to treat multidrug-resistant bacterial infections has been reconsidered in recent years [8]. Many clinical trials have shown that they have great potential, but how to prepare clinical standardized formulations for use in bacterial control and how to avoid the risk of phage-resistant bacteria and the transmission of genetic material still need to be resolved [9,10,11,12]. In addition, owing to the abundant prevalence of biofilms in hospital settings, a number of strategies have focused on developing efficient physical, chemical and biological methods for their prevention and disruption, such as bacteriocin- and essential oil-coated surfaces and using small molecules to interfere with the expression of virulence genes, including those necessary for biofilm formation [13,14,15,16]. Furthermore, it is noteworthy that the previously formed biofilm should be removed by degrading the extracellular matrix or eradicating bacteria inside the structure to reduce the nosocomial infection rate. Alternative strategies to antibiotics such as bacteriophage-derived endolysins, peptidoglycan degrading enzymes and polysaccharide depolymerase proteins have been proposed [17,18,19,20,21,22,23]. Lu and Collins found that a depolymerase-expressing engineered phage could degrade *Escherichia coli* biofilms to a significant degree compared with that of the nonengineered control phage [24]. Lood et al. explored the ability of phage endolysin (PlyF307) to reduce planktonic and biofilm *A. baumannii* both in vitro and in vivo [25], whereas another group reported that Lys ABP-01 activity could hydrolyze the cell wall and synergistically interact with colistin [26]. *Acinetobacter* podophage Petty elicits a novel lysis mechanism and tail-associated depolymerase activity capable of degrading CPS [27]. Phage-encoded capsule depolymerases Dpo48, Dp49 and B9gp69 also exhibit attractive antimicrobial activity against *A. baumannii* infections [28,29,30].

We previously identified and characterized one phage, φAB6, infecting *A. baumannii*, which exhibits plaques surrounded by a halo-zone. φAB6 also has a tailspike protein (TSP) containing a pectin lyase domain, which shows host specificity and depolymerase activity [31]. Furthermore, crystal structures of an N-terminally-truncated TSP revealed a trimeric β-helix architecture that bears inter-subunit carbohydrate-binding grooves and the structures suggest that pseudaminic acid in the substrate is an important recognition site for TSP [32]. In this study, to develop an effective anti-biofilm/antimicrobial agent as an alternative approach to reduce clinical infections, the anti-biofilm and antibacterial activity of purified TSP was investigated.

## 2. Results

### 2.1. Recombinant TSP Protein Showed CPS Degrading Activity against A. baumannii

**Figure 1:**
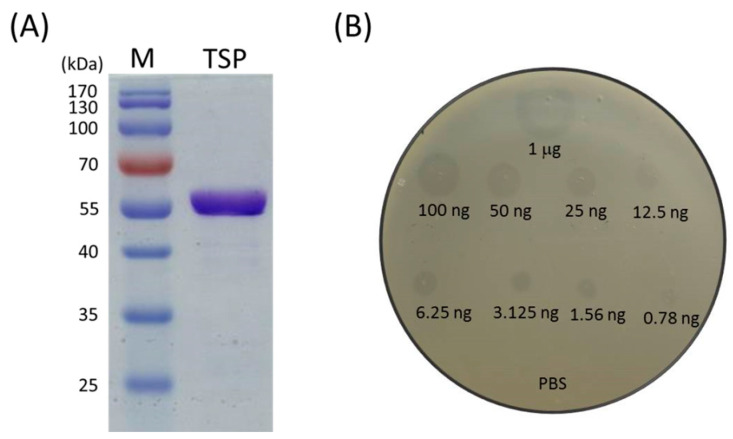
Tailspike protein (TSP) overexpression and activity assay. (**A**) Sodium dodecyl sulfate-polyacrylamide gel electrophoresis (SDS-PAGE) analysis of Ni-NTA His∙Bind^®^ Resin affinity column purified TSP. The protein sample was run on a reducing 10% gel. Lanes: M, molecular weight markers; TSP, purified protein. (**B**) TSP activity against its host, Ab-54149. Serial dilutions of TSP were dropped on the host strain and 20 mM PBS was used as the negative control.

The recombinant TSP was cloned, overexpressed in *E. coli* and purified by a nickel affinity procedure. The purity of the recombinant TSP protein is shown in Figure 1A. The activity of TSP was tested on an Ab-54149 (the host of φAB6) lawn by spot assay with inhibition zone detection. The results showed that the recombinant TSP could generate a translucent spot resembling a halo and the depolymerase activity was dose-dependent, even as low as 0.78 ng (Figure 1B). Next, the spot test was also used to evaluate the specificity of the enzyme in two reference strains, ATCC 17,978 and ATCC 19606 and 121 clinical *A. baumannii* isolates. As we expected, since TSP is the tailspike protein of phage φAB6 and a phage exhibits host specificity, the enzyme also possessed host specificity. That is, TSP could only decompose the capsular polysaccharide of its φAB6 host. Among the 121 clinical isolates tested, 73% are hosts of φAB6, so TSP worked on the capsular polysaccharides of these hosts. However, the two reference strains were not sensitive to φAB6; there were no translucent spots on the two tested bacterial lawns, indicating that TSP cannot work on non-host capsular polysaccharides.

**Figure 2:**
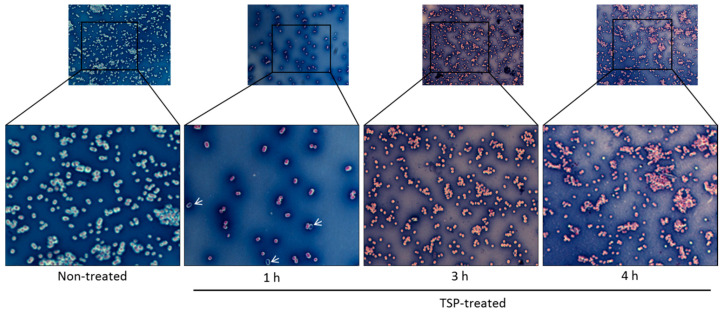
Maneval’s staining of extracellular material in overnight cultures of Ab-54149. From left to right: non-treated control group and cells treated with tailspike protein (TSP) for 1, 3 and 4 h at 37 °C. A magnification of the figure allows for the comparison of the presence/absence of polysaccharide matrix, with the absence represented by a white halo surrounding the cell. The white arrows show the remaining capsular material devoid of bacteria after 1 h of treatment.

Qualitative analysis of the potential of TSP to degrade the extracellular material formed by Ab-54149 in liquid culture was done by Maneval’s staining and light microscopy (Figure 2). Overnight cultured cells were incubated with 100 ng of TSP for 1, 3 and 4 h at 37 °C. Staining assays indicated that non-treated cells were surrounded by a thin layer of negatively stained capsular material. By contrast, with increasing TSP treatment time, the capsule gradually decreased or was drastically disrupted and remaining capsular material devoid of bacteria was also observed after 1 h treatment.

### 2.2. TSP Protein Can Remove and Inhibit A. baumannii Biofilm Formation

**Figure 3:**
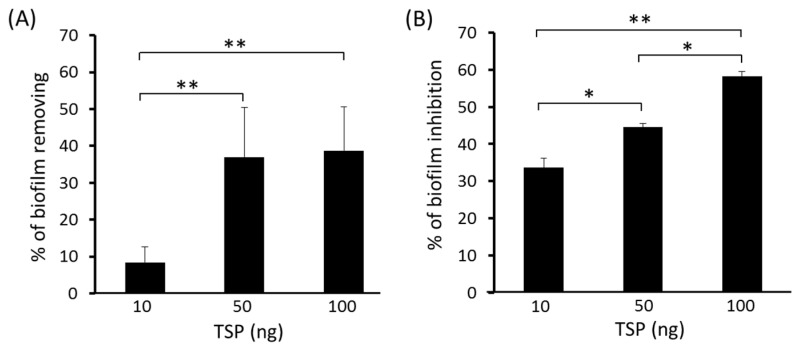
Biofilm removal (**A**) and inhibition activity (**B**) of tailspike protein (TSP) against Ab-54149 in microtiter plate assays. Values are the average of triplicates ± standard deviation (SD). Statistical significance was analyzed by two-tailed Student’s *t*-test (* *p* < 0.05, ** *p* < 0.01).

To test the ability of TSP to disperse *A. baumannii* biofilms, different amount of TSP (10, 50 and 100 ng/well) were incubated with the 48 h-old biofilms for 4 h. The sessile cells removing percentage of the 50 and 100 ng TSP groups were greater than that of the 10 ng TSP group. TSP removal of the biofilm structure from strain Ab-54149 was dose-dependent (Figure 3A). Additionally, to test the ability of TSP to inhibit biofilm formation, TSP (10, 50 and 100 ng/well) were co-cultured with host cells. The inhibition efficiencies of the three groups containing TSP were 30 to 60% (Figure 3B), indicating that TSP prevented biofilm formation and exhibited dose-dependent activity over 24 h.

**Figure 4:**
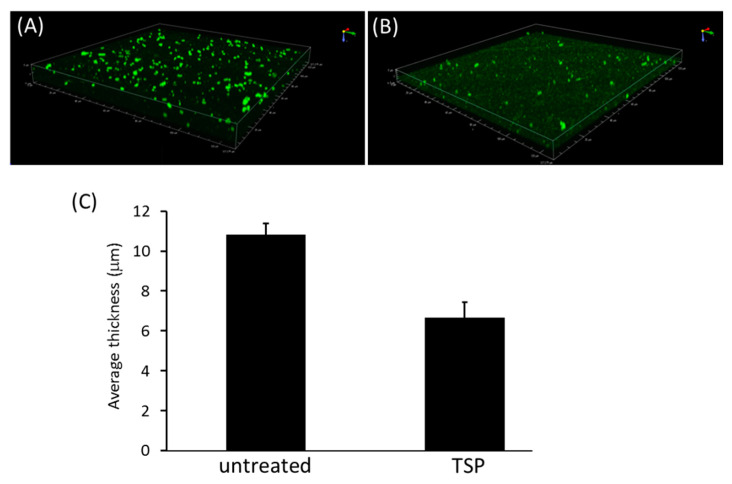
Reduction of Ab-54149 biofilm thickness by tailspike protein (TSP). Representative confocal microscope images of Ab-54149 showing biofilm architecture established after 48 h of incubation at 37 °C (**A**) and following 4 h treatment with 100 ng TSP (**B**) and quantification of both biofilm thicknesses (**C**). The bar graph represents the mean ± SD. Statistical significance was analyzed by a two-tailed Student’s *t-*test (*p* < 0.05). The biofilm spatial characteristic was determined by ISA confocal analysis software.

To evaluate biofilm dispersal by TSP, three-dimensional reconstruction architecture of *A. baumannii* biofilms after TSP treatment were generated from recorded z-stacks of confocal microscope images (Figure 4). The TSP treated groups had less biofilm thickness adhering to the slide than the untreated group.

### 2.3. TSP Increased Cell Membrane Permeability and Caused Morphological Changes in A. baumannii

**Figure 5:**
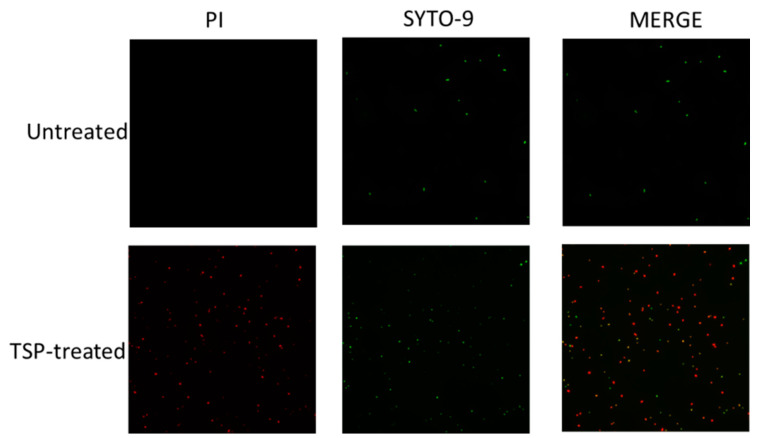
Bacterial membrane integrity was disrupted by tailspike protein (TSP). Confocal laser microscopy images of Ab-54149 (upper row) and Ab-54149 cells treated with TSP for 4 h (lower row) and stained with SYTO-9 and propidium iodide (PI) dyes. Green fluorescence indicates unaltered membrane integrity. Red fluorescence indicates membrane disintegration (permeable membrane).

To understand the effect of TSP on the bacterial cytoplasmic membrane, a Live/Dead BacLight^TM^ Bacterial Viability Kit was used to check the membrane integrity of TSP-treated and untreated *A. baumannii*. This kit is based on a combination of two nucleic acid-specific dyes, SYTO-9 and propidium iodide (PI), to distinguish between live and dead cells based on membrane integrity. SYTO-9 is a small molecule that can pass through intact plasma membranes and bind to DNA emitting green fluorescence (at 530 nm upon excitation at 488 nm), whereas PI only enters cells with compromised membranes and emits red fluorescence (at 620 nm upon excitation at 488 nm) by displacing SYOT-9. Thus, viable cells will be seen as green and dead as red. After co-staining with SYTO-9 and PI, the cells were observed by fluorescence microscopy. As shown in Figure 5, the untreated *A. baumannii* cells showed only green fluorescence, indicating that all cells were alive. In contrast, most cells treated with TSP showed red fluorescence, indicating membrane damage. The results revealed that the bactericidal effect of TSP on *A. baumannii* was directly through membrane permeabilization and damage.

**Figure 6:**
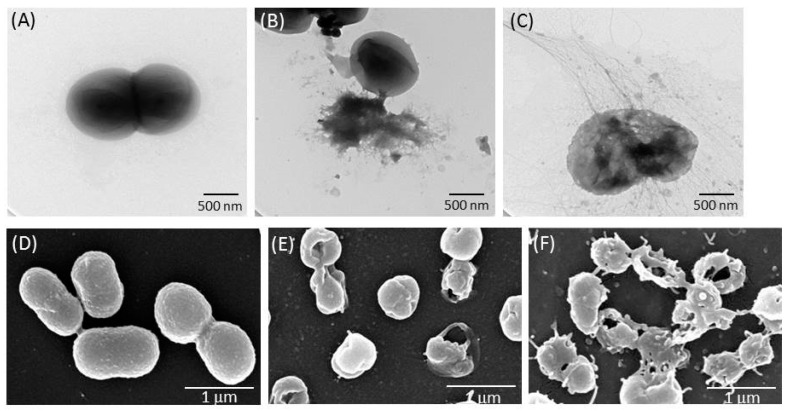
Transmission electron microscope (TEM) and scanning electron microscopy (SEM) analysis of tailspike protein (TSP)-treated Ab-54149. Untreated bacterial morphology was intact (**A**,**D**). Lysed cells were observed after treatment with TSP (100 ng) for 4 h (**B**,**E**), whereas most of the cells were lysed after 1 h of phage φAB6 infection (**C**,**F**).

Transmission electron microscopy (TEM) and scanning electron microscopy (SEM) observations were used to visualize the distinct morphological changes of the bacterial membranes treated with TSP. The results showed noticeable differences in the membrane morphology of the untreated and treated *A. baumannii*. TEM revealed a destroyed membrane and a few blebs in the treated cells. SEM revealed that the untreated bacterial membranes were smooth and intact, but treatment with 100 ng of TSP caused a leaky, wrinkled cell surface similar to phage φAB6-treated cells, indicating the loss of membrane integrity and cell lysis (Figure 6). These data suggest that TSP interacts with the membrane of *A. baumannii,* resulting in pore formation and leading to cell death.

**Figure 7:**
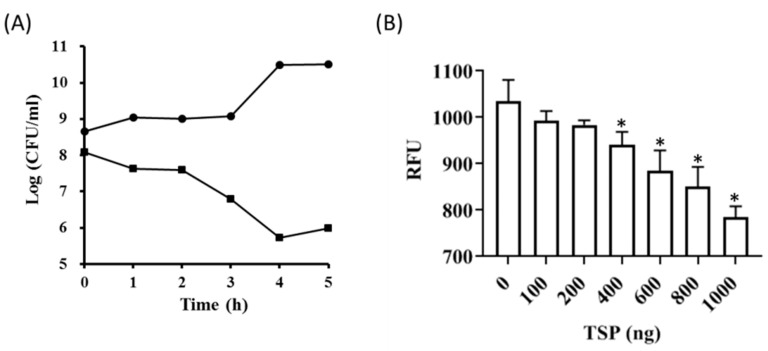
Cell viability and metabolic activity assay after tailspike protein (TSP) treatment. (**A**) Number of viable TSP-treated bacteria (ν) and untreated bacteria (λ) determined at the indicated time points. (**B**) PrestoBlue reagent was used to monitor the metabolic activity of TSP-treated cell. Data were collected from three independent experiments and expressed as mean ± SD. The readings were made in absorbance units in fluorescence units (560 nm excitation/590 nm emission). The asterisk (*) represented a statistically significant (*p* < 0.01) difference between the groups with TSP and without TSP treatment.

To evaluate the effect of TSP, cell viability was performed by counting the colony forming numbers at each indicated time point after plating on LA plates and metabolic activity was detected by the resazurin-based PrestoBlue™ Cell Viability Reagent. The results showed that host treatment with TSP produced a time- and dose-dependent decline in survival rate (Figure 7).

### 2.4. Inhibition of A. baumannii Cells Attached to TSP-Treated Foley Catheter Sections In Vitro

**Figure 8:**
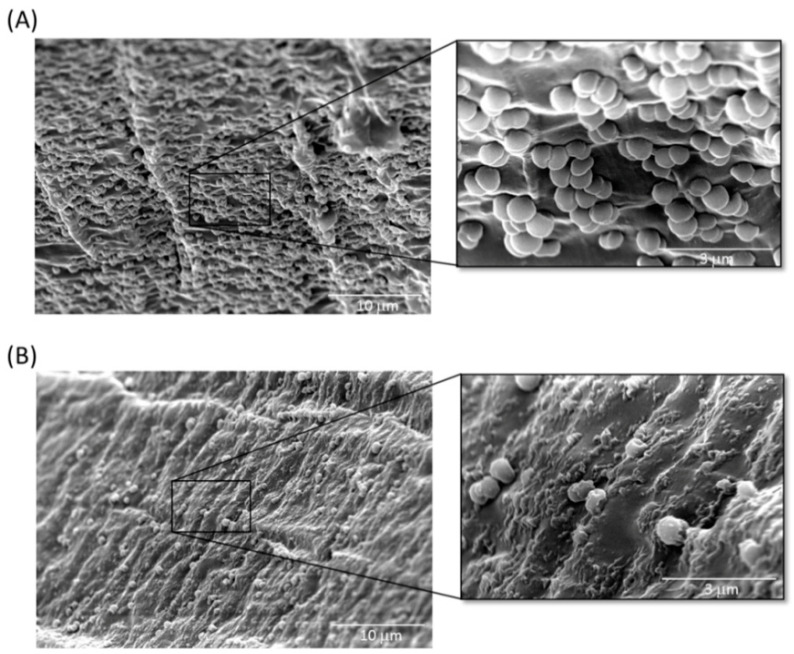
Scanning electron microscopy (SEM) images of the inhibition of *A. baumannii* cells attached to tailspike protein (TSP)-treated Foley catheter sections. (**A**) Surface of a section of the Foley catheter after biofilm formation by *A. baumannii* for 24 h. Attached cells are clearly visible in large quantities on the surface. (**B**) Surface of a section of the Foley catheter treated with TSP for 4 h after biofilm formation by *A. baumannii*.

Indwelling medical devices may become colonized with microorganisms, resulting in the formation of microbial biofilms and biofilm-associated microorganisms that are tolerant to antimicrobial agents, can evade the host immune system and can act as a nidus for infection. As reported by many investigators, the ability of *Acinetobacter* to form biofilms on abiotic surfaces increases the potential for bacteria colonization on hospital equipment and indwelling medical devices, such as urinary catheters, central venous catheters and endotracheal tubes [33,34,35]. In this study, we used TSP to treat *A. baumannii*-adhered silicone Foley catheters and performed a visual examination by SEM imaging. As shown in Figure 8, biofilms formed extensively on the surfaces of untreated catheter segments, whereas the number of attached *A. baumannii* cells was considerably less in all fields of view after TSP treatment for 4 h.

### 2.5. Therapeutic Effect of Depolymerase in Zebrafish

**Figure 9:**
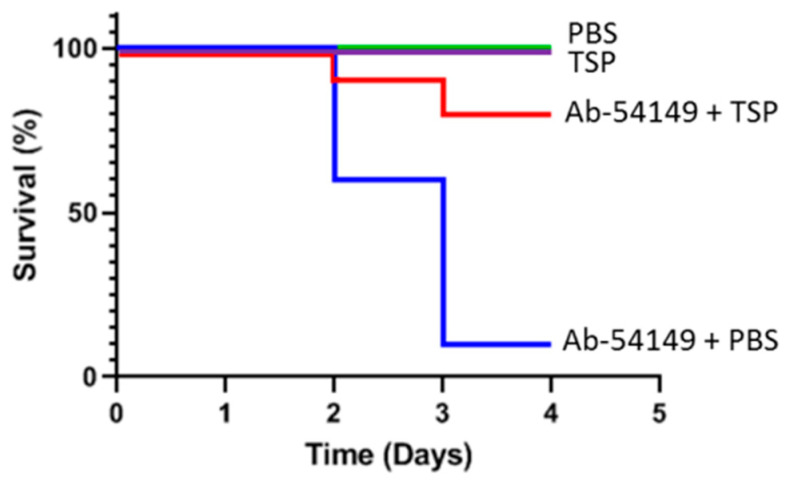
Therapeutic efficacy of depolymerase in zebrafish. The survival rates of zebrafish infected with Ab-54149 cells (1~4 × 10^7^ CFU inoculum) were measured after intraperitoneal injection of PBS (blue line) or tailspike protein (TSP; 20 μg/fish, red line) 30 min after challenge. Zebrafish were also injected with 20 μg of TSP or 20 μL PBS alone in the absence of bacterial infection as controls (green line). The *X*-axis represents the post-time of infection and the *Y*-axis represents the survival percentage of the zebra fish. Statistical analysis was performed using the Kaplan–Meier method. Treatment with TSP (*p* < 0.05; log–rank test) significantly increased the survival of zebrafish infected with strain Ab-54149 after TSP treatment within the 4-day period.

To evaluate the therapeutic effect of TSP depolymerase, zebrafish (n = 40) were administered PBS or 20 μL TSP (1 µg/µL) 30 min after challenge with Ab-54149. An equal dosage or volume of TSP or PBS, without Ab-54149 challenge, was administered as a control. The survival rate of the zebrafish infected with strain Ab-54149 was 10%, but in those treated with TSP it was 80%, within the monitored time period (0–4 days) (Figure 9). Furthermore, all of TSP-injected zebrafish survived, showing that TSP had no in vivo toxic effect.

## 3. Discussion

In the clinical environment, *A. baumannii* is one of the most alarming nosocomial Gram-negative pathogens with the ability to cause a variety of infections. Additionally, this species is susceptible to a limited range of antibiotics and becoming more resistant to several classes of antibiotics requiring the development of new antibacterial agents [1]. There is revived interest in phage therapy for controlling hard-to-treat bacterial infections at present. Although phages can be a promising alternative to antimicrobial agents, their success in the market will ultimately depend on the new regulatory framework and public acceptance. Therefore, phage-encoded depolymerase, a natural enzyme, might be more easily managed and used. Bansal and colleagues showed that *Aeromonas punctata* encoded depolymerase was able to degrade the capsule of *K. pneumoniae*, which resulted in restored sensitivity to gentamicin [36]. These data suggest that the use of depolymerases is an attractive approach for the treatment of biofilm-associated infections. To our knowledge, only a few studies have examined the efficacy of phage-derived depolymerase for combating *A. baumannii* infection [29,30,37]. However, *A. baumannii* has a strong ability to form biofilms. Biofilms can accumulate bacteria in the early stage of infection, making the bacteria difficult to be eradicated by the host’s immune defenses and antimicrobial agents and the bacteria that grow in biofilms are 10–1000 times more resistant to antibiotics than planktonic bacteria, thus increasing the severity of *A. baumannii* infection [38]. In addition, more than 90% of the biofilm is comprised of CPS that forms bulky extracellular structures covering the bacterial surface in layers; especially K2 capsular type has been associated with elevated antibiotic resistance [37]. Therefore, strategies that focused on developing new therapies that target the CPS will help combat bacterial biofilm infections. Many novel compounds are being developed with anti-biofilm activity that includes antimicrobial peptides, natural products, small molecules and polymers [39]. Phages are also being considered for use in treating biofilms as well as the use of phage-derived enzymes that degrade the extracellular matrix polymers, dissolving the biofilm [40]. There is great potential in these new approaches for use in treating chronic biofilm infections, in which capsular depolymerases attract most attention, because their use avoids some of the disadvantages, such as purification and bacterial endotoxin removal, of phages. It is known that the phage tail fiber or tail spike protein often functions as a polysaccharide depolymerase and targets specific capsule types for enhancing the antivirulence activity [41]. In *A. baumannii*, more than 128 types of capsule loci (KL type) have been identified, so even though many phages depolymerase functions have been studied [28,30,37,42,43,44], unlike broad-spectrum bactericidal antibiotics, they still target only a limited number of bacterial strains. Therefore, identification of novel depolymerases and evaluation of their ability to control *A. baumannii* infections is important.

Previously, we demonstrated that the TSP of *A. baumannii*-specific phage φAB6 was host determinant and possessed depolymerase activity, which can degrade bacterial CPS into simpler oligosaccharide units [31,32]. Base on BLASTp analysis revealed that φAB6 TSP was highly homologous to the protein encoded by *Acinetobacter* phage vB_AbaP_B3 (YP_009610379; 96% at the amino acid level). This, most likely, indicates that tailspikes of phages φAB6 and vB_AbaP_B3 can interact specifically with K2 type CPS [37]. Here, we determined the effectiveness of purified recombinant TSP against biofilms and bacteria in vitro and in vivo for the first time. The findings of this study include (1) φAB6-derived TSP can not only inhibit the formation of biofilms and degrade pre-exiting biofilms in vitro, but also destroy the integrity of bacterial membranes; (2) cloaca injection of 20 μg TSP depolymerase is sufficient to protect zebrafish from fatal infection; and (3) treatment of Foley catheter sections with TSP can inhibit colonization by *A. baumannii*. These results are similar to previous reports [28,29,45], indicating that the phage-derived depolymerase can remove the protective extracellular polysaccharides of bacteria and reduce the toxicity of the bacteria. Thus, the loss or modification of bacterial surface structures makes the bacteria less pathogenic and/or sensitizes them to some antimicrobials or host defenses, such as phagocytosis by macrophages and the bactericidal action of serum. In this study, we used a zebrafish infection model to test the therapeutic effect of the depolymerase 30 min after bacterial infection. This approach reflects the actual utility of TSP in clinical treatment. It is suggested that when depolymerase is applied in vivo, it reduces the capsular polysaccharide on the surface of bacteria, consequently decreasing virulence and possibly increasing immune components that participate in antimicrobial eradication [45,46]. Furthermore, the experimental design used here to examine the CPS degrading effect of TSP was different from other studies that also aim to determine the phage tailspike degrading activity on CPS; those studies usually purified bacterial CPS, then digested with phage depolymerases and the resulting digestion products were fractionated by gel-permeation chromatography to yield oligosaccharides [32,47] or by quantified the reducing sugars released from bacterial surface [28]. Here, we examined the morphological change of TSP-treated cells by TEM and SEM directly. It is surprising to show that TSP causes leakage and wrinkling of the cell surface, destroys the integrity of the bacterial membrane and causes cell death (Figure 5, Figure 6 and Figure 7). Therefore, phage depolymerase used in combination treatment therapy with other antibiotics has a higher impact on targeted multidrug-resistant bacteria [48]. The feasibility of rapid production and low development cost may be a good option for phage depolymerase application [49]. The major advantage of TSP depolymerase is an effective treatment approach for phage and multidrug-resistance of *A. baumannii*. Moreover, the combination therapy of TSP with other pre-existing treatment options opens an alternative approach to combat multidrug-resistant *A. baumannii*. Olsen and Schmelcher demonstrated the synergistic efficacy of the endolysin LysK and the poly-N-acetylglucosamine depolymerase DA7 static and dynamic (flow cell-based) biofilm removal against *Staphylococcus aureus* [50]. However, official guidelines regulating depolymerase applications as a pharmaceutical drug have not yet been established. The phage depolymerase also requires the exact identification of the host-specificity of the bacteria.

It is well known that biofilm is an important factor for virulence, so destroying the structure of biofilm can be the first step to prevent infection. Zelmer et al. [51] reported that removing the CPS from the bacterial surface by the phage-encoded enzyme endosialidase E, reduced the virulence of *E. coli* K1 by interrupting bacteria transit from the gut to the brain via blood circulation and prevented death from systemic infection in a non-invasive neonatal rat model. In addition, Darouiche et al. [52] showed that the synergistic effect of DispersinB^®^ and triclosan could reduce the colonization of *S. aureus* on catheters, indicating that the combined use of anti-biofilm/antibacterial agents provides an alternative strategy for the clinical prevention of medical device contamination. Our findings demonstrate that treatment of Foley catheter sections with TSP alone is sufficient to inhibit colonization by *A. baumannii*. Moreover, the results suggest that removal of the extracellular material of *A. baumannii* prevents bacterial adhesion to the medical-device surface.

## 4. Conclusions

TSP can prevent bacterial adhesion to the surface of medical-devices and shows a therapeutic effect in the treatment of *A. baumannii*-induced infections. The mechanism of TSP function appears to be via the removal of CPS, loss of integrity of the cell membrane and subsequent exposure of the underlying bacterium to immune attack. This study provides novel insights revealing that a phage-derived depolymerase not only targets the bacterial capsule polysaccharide, but can also destroy the integrity of the cell membrane. Moreover, TSP shows therapeutic potential for infections caused by *A. baumannii*, indicating that its effects are equivalent to antibacterial agents.

## 5. Materials and Methods

### 5.1. Bacterial Strains and Growth Conditions

The bacterial strains used for this study are described as follows. *A. baumannii* 54149 (Ab-54149) was isolated from the Buddhist Tzu Chi General Hospital, Hualien, Taiwan. For cloning and expression of TSP, *E. coli* TOP10 and *E. coli* BL21(DE3) (Invitrogen Life Technologies, Carlsbad, CA, USA) were used, respectively. *A. baumannii* and *E. coli* were grown in Luria-Bertani (LB) medium at 37 °C. All strains were stored at −80 °C until used.

### 5.2. Tailspike Gene Cloning, Expression and Purification

In order to delete N-terminal phage_T7_tail domain (PF03906), the DNA fragment encoding the amino acid 125–699 sequences of the φAB6 tailspike gene *orf40* (accession number YP_009288671) [31] was amplified from phage φAB6 genomic DNA by PCR using the primers NdeI_ABTF6_F (5′-CATATGAGTGAAGCTGCTGCTCAAGAGGCTG C-3′) and XhoI_ABTF6_R (5′-CTCGAGTTAACTCGTTGCTGTAAA TGC-3′). The underlined nucleotides indicate recognition sequences for NdeI and XhoI. The PCR fragment was digested with NdeI and XhoI, then ligated with the pET-28a expression vector (Novagen, Madison, WI, USA) with a C-terminal His×6 tag and transformed into *E. coli* BL21(DE3) cells. The cells carrying recombinant plasmids were selected on LB agar plates containing 1 μg/mL kanamycin (Sigma-Aldrich, St. Louis, MO, USA). The resulting pET-TSP plasmid was verified by DNA sequencing on a 3730XL system (Thermo Fisher Scientific, Wilmington, DE, USA) The recombinant isolates were cultured to the exponential growth phase and then induced with 0.1 mM isopropyl-β-D-thiogalactopyran- oside (IPTG; Sigma-Aldrich, St. Louis, MO, USA) and further grown for 16 h at 20 °C. The cells were harvested by centrifugation (7000× *g*, 4 °C, 10 min), resuspended in lysis buffer (50 mM NaH_2_PO_4_, 300 mM NaCl, pH 8.0), then broken by passage through the Constant Systems CF1 (Constant Systems Ltd., Low March, Daventry Northants, UK). The bacterial lysate was then centrifuged at 13,000× *g* for 10 min at 4 °C and the supernatant was passed through a 0.22 μm filter. The filtrate was loaded onto a Ni-NTA column (Qiagen, Valencia, CA, USA) according to the manufacturer’s instructions. The proteins were eluted with 5 volumes of imidazole-containing buffer (50 mM NaH_2_PO_4_, 300 mM NaCl, 250 mM imidazole, pH 8.0) via a step gradient to recover the purified TSP. The purified TSP was dialyzed against 20 mM PBS (4 L × 2) at 4 °C for 24 h and then lyophilized and stored at −20 °C before use. Protein concentration was estimated by measuring the absorption at 280 nm with a NanoDrop^TM^ 2000c (Thermo Fisher Scientific, Wilmington, DE, USA) and confirmed using a Bio-Rad Protein Assay Kit, which is based on the Bradford dye-binding method (Bio-Rad Laboratories, Inc., Irvine, CA, USA). The molecular weight and purity of TSP was determined by sodium dodecyl sulfate 10% polyacrylamide gel electrophoresis (SDS-PAGE; Thermo Fisher Scientific) followed by staining with Coomassie Brilliant Blue (Sigma-Aldrich).

### 5.3. Determination of TSP Activity against A. baumannii

Ab-54149, the strain used to propagate φAB6, was used to test the lytic activity of the TSP. For this purpose, a spot test was performed to observe the inhibition zone for susceptibility testing. In brief, LB agar was overlaid with 0.7% top agar inoculated with 200 μL of fresh log-phase bacterial culture. After drying, 2 μL of TSP dilution drops at 1 μg and 100, 50, 25, 12.5, 6.25, 3.125, 1.56 and 0.78 ng were spotted onto the surface of a double-layer agar plate. PBS was used as the negative control. After overnight incubation at 37 °C, the plate was checked for zones of inhibition.

For capsule staining, the cells were negatively stained with Maneval’s solution (5% acetic acid, 5% phenol, 10% FeCl_3_·6H_2_O and 0.05% acid fuchsin; Carolina Biological Supply Company, Burlington, NC, USA) and analyzed by microscopy [53]. Briefly, 1 μL of TSP (100 ng) was added to 100 μL of cells that had been cultured overnight and the reaction was incubated at 37 °C for 1, 3 and 4 h and then transferred to 10 μL of a 1% aqueous Congo red solution (Sigma-Aldrich) and mixed. The mixture was then spread across a glass slide to form a thin film and air-dried. Another 10 μL of Maneval’s solution was dropped across the glass slide, the Congo red background was acidified to blue and the cell color changed to purple. Capsules were negatively stained and appeared white under the light microscope (100×, oil; Nikon, Tokyo, Japan).

### 5.4. Membrane Permeability Assay

Cytoplasmic membrane damage was assayed with the LIVE/DEAD^®^ BacLight™ Bacterial Viability Kit (Thermo Fisher Scientific), which contained two dyes, SYTO-9 and propidium iodide (PI). Briefly, overnight culture was refreshed to OD_600_ ≅ 0.1 and then incubated at 37 °C until an OD_600_ ≅ 0.8, after which whole cells were collected by centrifuging at 5000× *g* for 3 min and the pellet was washed twice with PBS. The pellets were resuspended in TSP solution, while the control group was resuspended in PBS, then incubated for 4 h at 37 °C, centrifuged again and washed twice with PBS. Freshly prepared SYTO-9 and PI were added and incubated for 15 min in a dark room. Dye-stained samples were observed under fluorescence microscopy using a Nikon Eclipse 6600 (Nikon, Tokyo, Japan).

### 5.5. Cell Viability Assay

The effect of TSP on the viability of planktonic cells was monitored hourly by counting the viable cells on plates. TSP (100 ng/mL) was added to a conical flask containing 50 mL of LB, to which a 1% inoculum from an overnight culture was added. The flask was incubated at 37 °C with agitation. The control consisted of growth medium with bacterial inoculum, but without TSP. In addition, the metabolic activity of TSP-treated bacteria was assayed by PrestoBlue Cell Viability Reagent (Thermo Fisher Scientific), a commercially available, ready-to-use, water-soluble preparation. The procedures and viability calculation of bacterial cells strictly followed the instructions for the PrestoBlue assay. In brief, after 4 h of TSP incubation with log phase bacteria at 37 °C with shaking, aliquots (90 μL) of bacterial suspension were transferred to individual wells of a 96-well microtiter plate and the resazurin reagent PrestoBlue was added at a concentration of 10% (*v*/*v*). After mixing, the plates were incubated for an additional 30 min and the fluorescence intensity of each well was then measured using a plate reader with excitation filter at 560 nm and emission filter at 590 nm.

### 5.6. Microscopic Analyses of TSP-Treated A. baumannii

For TEM observation, Ab-54149 (3 × 10^8^ CFU/mL) was incubated at 37 °C with 100 ng of TSP for 4 h. The sample was centrifuged at 8000 rpm and the pellet was washed with deionized water. An aliquot of the resuspended sample (10 μL) was deposited on a 400-mesh carbon-coated grid, stained with 2% uranyl acetate and examined using a TEM (H-7500; Hitachi).

For SEM observation, the cells were grown on a plastic round coverslip placed in a 24-well microtiter plate for 48 h at 37 °C before adding TSP (100 ng) for an additional 4 h. The wells without TSP were used as the control. After treatment, the coverslips were washed twice with PBS, fixed with 2.5% (*w*/*v*) glutaraldehyde in 0.1 M cacodylate buffer and post-fixed in aqueous 1% osmium oxide. The samples were carefully rinsed twice with PBS, dehydrated using a graded ethanol series (50, 70 and 95%) and chemically dried in 100% hexamethyldisilazane (Sigma-Aldrich). Dried and gold-coated samples were observed under a SEM (S-4700; Hitachi, Tokyo, Japan).

Confocal laser scanning microscopy (CLSM) was used to evaluate the three-dimensional biofilm structure in the presence or absence of TSP-treatment. Biofilms were grown on coverslips as described above. After incubation, the coverslips were gently washed with PBS and stained with wheat germ agglutinin-Alexa Fluro (WGA-AF), which bound to bacterial extracellular polymeric substances (green fluorescence), for 2 h in a dark room. The excess stain was washed off and the stained coverslips were visualized using CLSM (Leica TCS SP2, Leica Microsystems GmbH, Wetzlar, Germany).

### 5.7. Biofilm Inhibition and Removability Assay

Quantitative estimation of biofilms formed by *A. baumannii* strains was assessed in 96-well polystyrene microtiter plates using the crystal-violet staining method described previously [52], with some modifications. To analyze the ability of TSP to inhibit biofilm formation, the tested bacterial cells were grown in LB at 37 °C overnight. The cultures were then harvested by centrifugation (5000× *g*, 15 min at 4 °C) and the pellet was rinsed with PBS. The washed bacterial cells were then resuspended in LB to approximately 5 × 10^7^ CFU/mL. TSP solution was then mixed with the bacterial inoculum to reach a final 10, 50 and 100 ng in 200 μL suspension. Wells of a non-adherence, sterile 96-well flat-bottomed plate were filled with 200 μL suspension. The control, untreated group consisted of bacterial suspension in PBS. The plates were covered and aerobically incubated for 24 h at 37 °C. Afterward, the content of each well was aspirated, the well was rinsed five times with 250 μL of sterile PBS, aspirated again and then left to dry. Subsequently, the samples in each well were stained for 5 min with 0.2 mL of 2% crystal violet (Sigma-Aldrich). Excess stain was rinsed off by inserting the plate under running tap water. After staining, the dye was then decanted and each well was washed twice with PBS and dried. Finally, 200 µL of 95% ethanol was added to each well and the OD was measured at 570 nm using a Microplate Photometer (Thermo Fisher Scientific). Inhibition efficiency was shown as a percentage and calculated as follows: [1 − (ODtreated/ODuntreated)] × 100%.

To test the ability of TSP to eradicate the formed biofilms, bacterial biofilms were developed in flat-bottomed 96-well plates for 48 h at 37 °C. After biofilm growth, the wells were rinsed with PBS to remove non-adherent cells. The biofilms in each well were subsequently treated with PBS (untreated control) or TSP (10, 50, or 100 ng) for 4 h at 37 °C. Afterward, a quantitative assessment of any remaining biofilm was carried out as described above. Degradation efficiency was shown as a percentage and calculated as follows: [1 − (ODtreated/ODuntreated)] × 100%.

### 5.8. SEM of Catheter Cross Sections

Segments (1 cm long) of silicone Foley catheter were immersed in 2 mL of *A. baumannii* culture and a biofilm was allowed to develop on segments by incubating at 37 °C (non-shaking) for 7 d using a fed batch culture method where the growth medium was replaced with fresh medium every 24 h. After 7 d, the catheter segments were washed once with 2 mL sterile PBS to remove the loosely attached planktonic bacterial cells, followed by adding TSP (100 ng) or PBS (control) for 4 h. After treatment, samples were washed twice with PBS, then fixed in 2.5% glutaraldehyde in PBS at 4 °C overnight. After three washes with PBS, samples were post-fixed with 1% osmium tetroxide for 1–2 h, then underwent step-wise dehydration in increasing percentages of ethanol from 25% to 100%. The dehydrated samples were dried with CO_2_ in a critical point drier prior to mounting on stubs and sputter coating with a thin gold layer. The coated samples were visualized under a SEM.

### 5.9. TSP Treatment for Zebrafish Infections

The zebrafish (*Danio rerio*) lines used were the wild type AB variety; they were maintained in the Tzu Chi University FishCore facility according to standard protocols. Mixed male and female populations of zebrafish were kept in 9 L tanks at 28 °C and maintained in a 14 h light/10 h dark cycle.

The zebrafish were grouped as follows (40 per group). One group was anesthetized with 0.2% tricaine and injected with 20 μL (1~4 × 10^7^ CFU) of *A. baumannii* strain Ab-54149 suspended in PBS through the cloaca with an insulin needle. The other group was infected with the same dose of Ab-54149 strain and injected with a dose of 20 μg (20 μL) TSP into the cloaca 30 min later. In addition, to assess potential acute toxicity from the TSP, zebrafish were injected with 20 μg (20 μL) of TSP or an equal volume of PBS alone in the absence of bacterial infection. After treatment, the zebrafish were transferred to separate tanks and monitored at least twice daily for 4 days. The numbers of surviving fish at day 4 were plotted and Fisher’s Exact Test was used to evaluate the therapeutic efficacy of TSP. Using SPSS software, Kaplan–Meier survival curves [53] were plotted to show the cumulative probability of survival over the 4-day period, using the Log Rank test or generalized Wilcoxon test for statistics. The laboratory protocol was approved by the Institutional Animal Care and Use Committee of Tzu Chi University (IACUC Approval NO.: 109018)

### 5.10. Statistical Analyses

All experiments were performed in triplicate and the values are expressed as mean ± standard deviation (SD). Differences between two experimental groups were analyzed with the two-tailed Student’s *t-*test. A *p*-value < 0.05 was considered statistically significant.

## Data Availability

Not applicable.
